# Supplementary material for: Micro-occurrence characteristics and charging mechanism in continental shale oil from Lucaogou Formation in the Jimsar Sag, Junggar Basin, NW China
Source: PLoS One. 2024 Feb 5;19(2):e0297104. doi: 10.1371/journal.pone.0297104 (PMC10843125; doi:10.1371/journal.pone.0297104)
Supplement: Attachment — Submitted filename: Response to Reviewers.docx [file pone.0297104.s002.docx]

**Response to the third round of editorial department requests**.

1. We note that in Figures 5 and 8, there appear to be software logos in your images, which cannot be published under PLOS ONE's license. Please remove these logos and resubmit these images.

Response: Thank you for the reminder. We removed the software logos in Figure 5 and Figure 8 and re-uploaded them.

2.We also note that Figures 1 and 2 appear to be missing. Please either resubmit these two figure files or clarify why they have been removed.

Response: We apologize for our oversight. We have re-uploaded Figures 1 and 2 and have confirmed that there are no omissions this time.

**Response to the second round of editorial department requests**.

1. Please state what role the funders took in the study. If the funders had no role, please state: "The funders had no role in study design, data collection and analysis, decision to publish, or preparation of the manuscript."

If this statement is not correct you must amend it as needed.

Please include this amended Role of Funder statement in your cover letter; we will change the online submission form on your behalf.

Response: The funders had no role in study design, data collection and analysis, decision to publish, or preparation of the manuscript.

2. If there are no restrictions, please upload the minimal anonymized data set necessary to replicate your study findings to a stable, public repository and provide us with the relevant URLs, DOIs, or accession numbers. For a list of recommended repositories, please see

https://journals.plos.org/plosone/s/recommended-repositories. You also have the option of uploading the data as Supporting Information files, but we would recommend depositing data directly to a data repository if possible.

Response: We uploading the data as Supporting Information files. We have provided the minimal anonymized datasets for Figures 3, 6, 7, 9, 11, 12 and 14, while Figures 1, 2, 4, 5, 8, 10 and 13 do not contain any data or the data is already presented in the text or in the corresponding references.

3. Where did the authors obtain the satellite images in Figures 4 and 8? Please provide specific links to where you found these resources to create these images and any relevant copyright information.

Response: Please note that Figures 4 and 8 are not satellite images! These are backscattered images of the sample surfaces taken under high-resolution scanning electron microscopy, used to observe the pore structure, mineral types, and distribution of hydrocarbon substances in the samples. These images were captured in our laboratory, and we hold full copyright for them.

**Response to the first round of review experts and editorial department requests:**

**Requests from the editor:**

1. Please ensure that your manuscript meets PLOS ONE's style requirements

Response: We have diligently reviewed the style requirements of PLOS ONE and made the necessary revisions to ensure that our manuscript complies with these standards.

2. NMR spectroscopy data

Response: Our NMR spectroscopy data pertains to complex mixtures found within rock samples. These mixtures consist of various large organic molecules that form intricate organic compositions. They significantly differ from conventional NMR testing targets, making it challenging to provide specific compound names or chemical structures. If you have any further inquiries or require additional details regarding the NMR data, please do not hesitate to ask, and we will provide the necessary information. Thank you for your understanding.

3. In your Methods section, please provide additional information regarding the permits you obtained for the work.

Response: We have included additional information in the Methods section regarding the permits obtained for the work. Our laboratory is fully accredited and authorized for the research conducted in this study. If you require any further details or have specific questions about the permits, please feel free to ask, and we will provide the necessary information. Thank you for your attention to this matter.

4. Please state what role the funders took in the study.

Response: The funder for this study, Professor Zhanghua Lou, provided financial support for the research and was actively involved in the manuscript's development and revision.

5. Data Availability statement

Response: This is an original research article and will be used in the first author's doctoral dissertation as a part of their Ph.D. thesis. Therefore, regarding the issue of data sharing in the article, we would appreciate further communication with your esteemed journal.

6. ORCID iD

Response: We have successfully updated our ORCID information. Thank you for the guidance, and our ORCID iD is now validated in Editorial Manager. We appreciate your assistance.

7. We note that Figure 1 in your submission contain map images which may be copyrighted.

Response: We appreciate your attention to Figure 1 in our submission. It's important to clarify that the map images included in Figure 1 do not raise any copyright concerns. The contents of this figure are publicly available and can be referenced without any issues. The structural zoning map of the Junggar Basin is a publicly available resource published on the following website: https://geocloud.cgs.gov.cn/commonsearch/search/detail?globalId=cpgl_dzcp_a8ae3ae967cf4c7b9a345e4de37cd01b&networkType=extranet&table_name=cpgl_dzcp&isAccurate=false&keyword=%E5%87%86%E5%99%B6%E5%B0%94. The structural maps of the Jimsar Sag are well-documented in numerous references, including those by Si KL (2015), Ma K (2017), Guo HP (2023), and Yin SL (2022), among others. Their use in our publication is in compliance with standard citation practices. We are confident that the use of these maps is within acceptable copyright guidelines. Thank you for your understanding.

8. We note that Figure 9 in your submission contain copyrighted images.

Response: Thank you for bringing this to our attention. We want to clarify that the images in Figure 9 are original photographs taken by our team and do not involve any copyright issues. These electron microscopy images are our own work, and we hold the rights to use them in our publication without any copyright concerns.

**Comments from the Reviewer #1:**

1) As for the title, "development response" is rarely discussed in this paper, and it is suggested to change it to "charging mechanism" or “accumulation mechanism”. In addition, the contents of section 4.6 do not belong to discussion, and are more suitable as prospects in the conclusion.

Response: Thank you. In response to your valuable suggestion, we have made the recommended modifications. Firstly, we have revised the title to better align with the content of the paper. "Development response" has been changed to "charging mechanism" to accurately reflect the focus of the study. Additionally, we have addressed the concern regarding the placement of content in section 4.6. We have relocated this section from the discussion to the conclusion, where it is now presented as a new chapter labeled "Prospects."

2) The abstract does not effectively summarize the manuscript. The abstract does not make clear what problems exist and what the significance of the study is. Suggest rewriting. There is no direct correlation between this research and the oil development raised by authors.

Response: We appreciate the feedback provided. In response to your comments, we have thoroughly reviewed and amended the abstract to more effectively summarize the manuscript. This revision places a stronger emphasis on the issues addressed in our research and the significance of our findings. While the study may not directly guide shale oil development, it does offer valuable insights into the micro-occurrence characteristics of shale oil, shedding light on its microscopic attributes, mobility, and potential applications in refining extraction processes. We trust that these revisions will better align the abstract with the core aspects of our study.

3) As for the introduction, three questions need to be answered clearly in the introduction: what research question(s) do the authors address? Do they make a good argument for why a question is important? What methods do the authors use to answer the question? The authors only raised one existing question in the introduction, that is, the extraction standard is not uniform. It is suggested to rewrite the introduction.

Response: Thank you for your valuable guidance. We have carefully restructured the introduction in response to your recommendations. The revised introduction now explicitly addresses the research questions we are investigating, provides a compelling argument for the significance of these questions, and thoroughly explains the methods employed to address these inquiries. We believe this restructured introduction successfully aligns with your feedback, and we appreciate your assistance in enhancing the clarity of our manuscript.

4) The author provides a lot of figures with simple figure name. The information and conclusions of these figures should be described in detail in figure caption to facilitate readers' better understanding. Some of the information in the figure is not even found in the text; for example, the formation abbreviation and oil saturation in Figure 1 are not stated.

Response: Thank you very much for your suggestion. We have thoroughly reviewed all figure captions and made modifications and additions to each of them, providing more detailed information related to the figures. We believe that these revisions will significantly enhance readers' understanding of the figures.

5) The XRD results and figures in Chapter 3 should be transferred to Chapter 4. The authors are advised to add the duration range and temperature of extraction. It is recommended to supplement the instruments, experimental parameters and laboratories for all experiments.

Response: Firstly, we acknowledge the suggestion to move the XRD results from Chapter 3 to Chapter 4. However, upon careful consideration, we have decided to remove the XRD results from the manuscript. After a comprehensive review, we found that the XRD results played a limited role in the paper and did not significantly contribute to the overall content.

Secondly, we have addressed the recommendation by adding details about the experimental instruments, parameters, and laboratory information for all experiments. This addition should provide readers with a clearer understanding of the experimental setup and conditions.

6）It is suggested that the authors divide the results and discussion in Chapter 4 into two chapters. Given that the rock types of the Lucaogou Formation are very complex and diverse, the number of samples collected by the authors is slightly insufficient (only two), which may lead readers to question the statistical significance of the results. In addition, the authors do not discuss how representative the sample is to the stratum, and whether only a few samples can be representative of the whole stratum.

Response: Thank you for your suggestion. After careful consideration, we have decided to keep the results and discussion in Chapter 4 together, as this structure aligns better with the overall flow of the paper.

Regarding the issue of sample quantity and representativeness, we have provided additional explanations in the "Samples and Experiments" section. While we acknowledge that the number of samples is limited, they do represent the primary oil-bearing layers and lithologies of the Lucaogou Formation, making them reasonably representative. Through the study of these samples, we aim to contribute to a comprehensive understanding of the micro-occurrence characteristics of shale oil in the Lucaogou Formation.

7) The historical process of oil charging is not clearly described. The source of the buried history data needs to be explained. There should be an obvious uplift denudation of K2-E, leading to large-scale denudation of the upper part of Lusaogou Formation in Jimsar Sag.

Response: Thank you for your suggestion. We have revisited the description of the historical process of oil charging and provided an explanation regarding the source of buried history data.

In reference to your comment about the "obvious uplift denudation of K2-E," we may need further clarification on this point as it wasn't entirely clear. However, we believe that this should not significantly impact the overall micro-filling process of shale oil within the context of the charging history. If you have any further questions or clarifications, please feel free to let us know, and we will address them accordingly.
